# Developing Fast, Red-Light Optogenetic Stimulation of Spiral Ganglion Neurons for Future Optical Cochlear Implants

**DOI:** 10.3389/fnmol.2021.635897

**Published:** 2021-03-11

**Authors:** Antoine Tarquin Huet, Tobias Dombrowski, Vladan Rankovic, Anupriya Thirumalai, Tobias Moser

**Affiliations:** ^1^Institute for Auditory Neuroscience and InnerEarLab, University Medical Center Göttingen, Göttingen, Germany; ^2^Auditory Neuroscience and Optogenetics Laboratory, German Primate Center, Göttingen, Germany; ^3^Department of Otolaryngology, Head and Neck Surgery, St. Elisabeth Hospital, Ruhr University Bochum, Bochum, Germany; ^4^Collaborative Research Center 889, University of Göttingen, Göttingen, Germany; ^5^Restorative Cochlear Genomics Group, Auditory Neuroscience and Optogenetics Laboratory, German Primate Center, Göttingen, Germany; ^6^Göttingen Graduate School for Neurosciences and Molecular Biosciences, University of Göttingen, Göttingen, Germany; ^7^Multiscale Bioimaging Cluster of Excellence (MBExC), University of Göttingen, Göttingen, Germany

**Keywords:** ear, hearing restoration, prosthetics, gene therapy, virus, cochlear implant, optogenetics

## Abstract

Optogenetic stimulation of type I spiral ganglion neurons (SGNs) promises an alternative to the electrical stimulation by current cochlear implants (CIs) for improved hearing restoration by future optical CIs (oCIs). Most of the efforts in using optogenetic stimulation in the cochlea so far used early postnatal injection of viral vectors carrying blue-light activated channelrhodopsins (ChRs) into the cochlea of mice. However, preparing clinical translation of the oCI requires (*i*) reliable and safe transduction of mature SGNs of further species and (*ii*) use of long-wavelength light to avoid phototoxicity. Here, we employed a fast variant of the red-light activated channelrhodopsin Chrimson (f-Chrimson) and different AAV variants to implement optogenetic SGN stimulation in Mongolian gerbils. We compared early postnatal (p8) and adult (>8 weeks) AAV administration, employing different protocols for injection of AAV-PHP.B and AAV2/6 into the adult cochlea. Success of the optogenetic manipulation was analyzed by optically evoked auditory brainstem response (oABR) and immunohistochemistry of mid-modiolar cryosections of the cochlea. In order to most efficiently evaluate the immunohistochemical results a semi-automatic procedure to identify transduced cells in confocal images was developed. Our results indicate that the rate of SGN transduction is significantly lower for AAV administration into the adult cochlea compared to early postnatal injection. SGN transduction upon AAV administration into the adult cochlea was largely independent of the chosen viral vector and injection approach. The higher the rate of SGN transduction, the lower were oABR thresholds and the larger were oABR amplitudes. Our results highlight the need to optimize viral vectors and virus administration for efficient optogenetic manipulation of SGNs in the adult cochlea for successful clinical translation of SGN-targeting gene therapy and of the oCI.

## Introduction

Sensorineural hearing impairment, the most common form of hearing impairment, is typically characterized by a dysfunction or loss of hair cells transducing sound signals and generating a neural code at their synapses with spiral ganglion neurons (SGNs). The etiology of sensorineural hearing impairment ranges from genetic defects to aging, external factors as noxious sounds, ototoxic antibiotics, and chemotherapeutic agents. Typically, disorders converge in a dysfunction and loss of sensory hair cells (see recent review in Kleinlogel et al., 2020). As of today, causal treatment options such as gene therapy or hair cell regeneration are not yet available for clinical use. Hence, hearing aids and cochlear implants have remained the leading means of hearing rehabilitation. The electrical cochlear implant (eCI) directly stimulates SGNs electrically (Zeng et al., 2008; Lenarz, 2018). eCIs partially restore hearing and enable open speech intelligibility to most of its ∼700,000 users, making the eCI the most successful neuroprosthesis. Nonetheless, hearing in more complex acoustic scenes such as speech in noise or music is impeded by the wide spread of electrical current from each electrode contact, thus recruiting a large portion of SGNs and limiting the maximum number of independent channels to ≤10 (Kral et al., 1998; Zeng and Galvin, 1999; Friesen et al., 2001; Middlebrooks, 2005).

Optical stimulation presents an attractive alternative, as light can be better confined in space (Richter and Tan, 2014; Kleinlogel et al., 2020). Photosensitization of neurons can be achieved by optogenetics, i.e., expressing Channelrhodopsins (ChRs) in the plasma membrane of neurons (Nagel et al., 2003; Boyden et al., 2005). Optogenetic stimulation of the SGNs was shown to activate the auditory pathway (Hernandez et al., 2014; Duarte et al., 2018; Keppeler et al., 2018; Mager et al., 2018; Wrobel et al., 2018; Dieter et al., 2019, 2020b; Keppeler et al., 2020; Thompson et al., 2020) and to restore hearing in various models of deafness (Hernandez et al., 2014; Mager et al., 2018; Wrobel et al., 2018; Keppeler et al., 2020). Recent studies, characterizing the cochlear spread of excitation by recordings from the inferior colliculus, validate the advantage of optogenetic SGN stimulation over conventional electrical stimulation (Dieter et al., 2019, 2020b; Keppeler et al., 2020). Introduction of a given opsin (i.e., a photosensitive protein) into neurons can be performed by viral gene transfer (for review, Kleinlogel et al., 2020). Wrobel et al. (2018) pioneered intramodiolar AAV- injection into the cochlea of adult gerbil to transduce SGNs with CatCh (i.e., Channelrhodopsin variant showing an enhanced Ca^2+^ permeability; Kleinlogel et al., 2011). This approach enabled optically evoked auditory brainstem responses (oABR) in 46% of the injected gerbils (Wrobel et al., 2018). The cochleae of responsive animals showed an average SGN transduction rate of ∼30% with relatively even distribution across all cochlear turns. The observed low transduction efficiency highlights the need to improve the optogenetic modification of adult SGNs for the development of the oCI toward clinical translation. Additionally, generating and characterizing ChRs appropriate for restoring sound encoding in SGNs is critical. The ideal candidate ChR (for reviews, see Jeschke and Moser, 2015; Dombrowski et al., 2019; Dieter et al., 2020a) should combine (*i*) large ion conductance: to confer high light sensitivity to SGNs via large photocurrents while keeping the number of expressed ChRs low; (*ii*) fast kinetics: to enable firing rates as found in physiology, ∼200–300 spike/s (Heil and Peterson, 2015; Huet et al., 2016) and phase-locking of firing up to ∼ few hundreds Hz of stimulation (Heil and Peterson, 2016); (*iii*) a red-shifted action spectrum for lowering the risk of phototoxicity (Kerstein et al., 2014) and for deeper light propagation (Jacques, 2013). The Chrimson (Klapoetke et al., 2014) variants f-Chrimson and vf-Chrimson, obtained from mutagenesis of helix 6, showed good photocurrents (f-Chrimson > vf-Chrimson), fast kinetics (τ_*off*_ at body temperature of 3.2 and 1.6 ms for f-Chrimson and vf-Chrimson, respectively), and a red-shifted action spectrum making them interesting candidates for the development of the oCI (Mager et al., 2018). Indeed, f-Chrimson was successfully expressed in SGNs of mice following an early postnatal viral cochlear injection and enabled SGN stimulation at few hundreds Hz at the population and single neuron levels (Mager et al., 2018). This study used early postnatal AAV2/6 injection into the scala tympani and achieved high SGN transduction rates in most injected mice (approximately 75% of SGNs transduced on average across all turns).

In order to further evaluate the potential of f-Chrimson for future optogenetic hearing restoration, we employed the Mongolian gerbil as a preclinical rodent model with a larger cochlea and a hearing frequency range more similar to humans than found in mice (Dieter et al., 2020a). Specifically, in order to bridge the successful early postnatal SGN transduction in mice (e.g., Duarte et al., 2018; Keppeler et al., 2018; Mager et al., 2018) and the less efficient SGN manipulation in the adult cochlea of gerbils (e.g., Wrobel et al., 2018; Dieter et al., 2019, 2020b) we compared early postnatal (P8) and adult (>8 weeks) cochlear administration of a potent AAV vector: AAV-PHP.B (Deverman et al., 2016) carrying f-Chrimson. Thereby, we aimed to distinguish the hypotheses that the difference in transduction efficiency between previous studies in mice and gerbils was (*i*) due to the age at AAV-administration or (*ii*) due to species differences. We compared scala tympani injections into the postnatal cochlea to intramodiolar injection into the adult cochlea, since AAV injection into scala tympani of adult gerbils was previously found to be rather inefficient (Wrobel et al., 2018) and intramodiolar injection of the early postnatal rodent cochlea seems intractable. We evaluated f-Chrimson expression in SGNs by recordings of optically evoked auditory brainstem (oABR) as well as by post-mortem immunohistochemistry to probe transduction rate and f-Chrimson expression in the SGN plasma membrane. Moreover, we analyzed adult injections with AAV2/6 at higher titer and compared it to AAV-PHP.B from the first set of experiments. Finally, we employed adult intramodiolar AAV2/6 administration at different speeds in order to assess the particular affection of transduction rate and pressure-induced SGN loss (Wrobel et al., 2018).

## Results

### Early Postnatal vs. Adult Optogenetic Manipulation of Spiral Ganglion Neurons via Cochlear Injection of AAV-PHP.B-f-Chrimson

**FIGURE 1:**
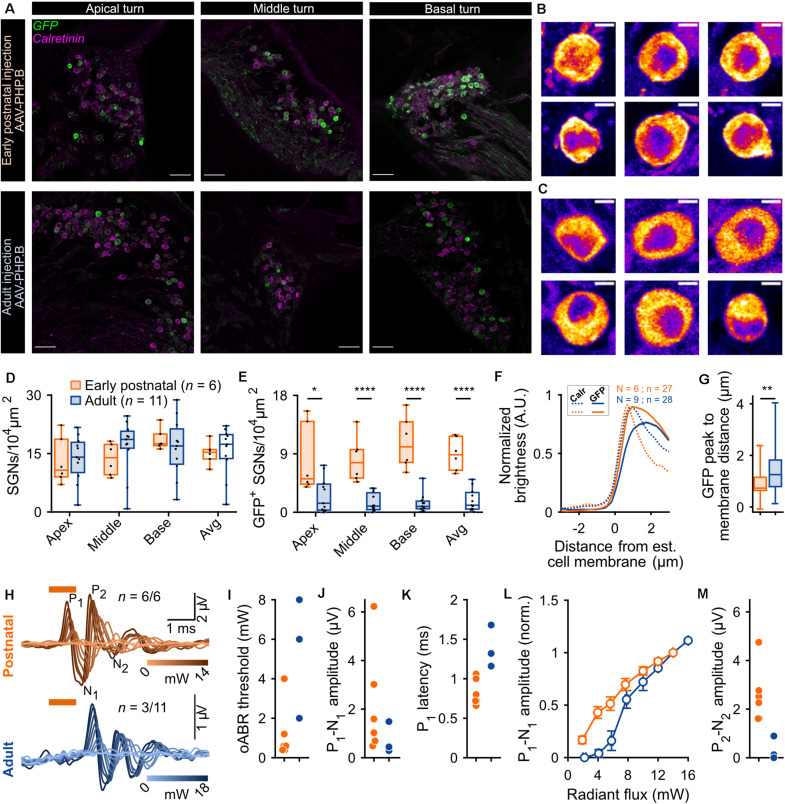
Comparison of f-Chrimson expression in spiral ganglion neurons (SGNs) and optically evoked auditory brainstem responses (oABR) between early postnatally and adult injected cochleae using AAV-PHP.B as a viral vector. **(A)** Representative maximum projection of confocal images obtained from immunolabeled mid-modiolar cochlear cryosections (cochlear apical, middle and basal turn) following early postnatal (P8, top, orange) or adult (>8 weeks, bottom, blue) cochlear pressure injection of AAV-PHP.B-f-Chrimson. GFP (green) marks transduced SGNs and calretinin (magenta) was used as a generic SGN marker (scale bar = 50 μm). **(B,C)** Magnified view on single SGN somata (from **A**) in the GFP channel from early postnatally **(B)** and adult **(C)** injected cochleae. The color code was changed to “fire” and dynamically adjusted to highlight in white the GFP peak expression (Scale bar = 5 μm). **(D,E)** SGN density **(B)** and density of GFP-positive SGNs **(C)** measured at the cochlear apical, middle and basal turn and average of the 3 turns for early postnatally (*n* = 6) and adult (*n* = 11) AAV-PHP.B-f-Chrimson injected cochleae. Wilcoxon rank sum test (*p ≤ 5 × 10^–2^, ****p ≤ 10^–4^). Box plots show minimum, 25th percentile, median, 75th percentile, and maximum with individual data point overlaid. **(F)** Normalized line profiles, from early postnatally (orange, *N* = 6 cochleae, *n* = 27 SGNs) and adult (blue, *N* = 9 cochleae, *n* = 28 SGNs) injected cochleae, were measured for calretinin (Calr, dashed lines) and GFP (plain lines) channels, normalized and aligned at 30% of Calretinin rise. **(G)** Quantification of the distance between the estimated cell membrane (i.e., 30% of Calretinin rise) and the peak of the GFP line profile from early postnatally (orange) and adult (blue) injected cochleae (data presented in D). Wilcoxon rank sum test (**p ≤ 1 × 10^–2^). Box plots show minimum, 25th percentile, median, 75th percentile, and maximum. **(H)** Largest recorded oABR of gerbils following early postnatal (top, orange) and adult (bottom, blue) injected cochleae for various light radiant flux (λ = 594 nm, pulse duration = 1 ms, repetition rate = 10 Hz). oABRs could be recorded in 6 out of 6 early postnatally injected gerbils vs. 3 oABRs out of 11 adult injected gerbils. The color scale encodes the radiant flux. **(I–K,M)** oABR threshold **(E)**, P_1_-N_1_ amplitude (at 14 mW, **F**), P_1_ latency (at 14 mW, **G**) and P_2_-N_2_ amplitude (at 14 mW, **I**) obtained from gerbils following early postnatal (orange) or adult (blue) cochlear AAV injection. **(L)** P_1_-N_1_ amplitude growth function of oABRs from early postnatally (orange) and adult (blue) injected gerbils. Amplitude were normalized relative to the amplitude at 14 mW. Average ± SEM.

Several studies aiming at optogenetic manipulation of SGNs were performed using AAV injection into the scala tympani of mice aged between 3 and 8 days (Duarte et al., 2018; Keppeler et al., 2018; Mager et al., 2018). This approach enabled oABRs in the majority of the injected animals (>90%) and achieved a high transduction rate across the 3 cochlear turns often exceeding ∼70% on average. In contrast, when directly injecting the AAV suspension into the scala tympani of adult gerbils (>8 weeks), little or no transduction of SGNs was found (Wrobel et al., 2018 and Supplementary Figure S1A). Considering lower SGN accessibility for AAVs from scala tympani of the fully ossified mature cochlea, direct intramodiolar AAV-administration toward the SGN somata via a minute drilled hole was implemented. Still, only 46% of the intramodiolarly injected adult gerbils showed oABRs. In those animals an average transduction rate of ∼30% of SGNs was found across the cochlear turns (Wrobel et al., 2018). To address the reasons for the different outcome of transduction for postnatal AAV-injection into the mouse cochlea and for AAV-injection into the adult gerbil cochlea, we established the postnatal AAV injection (8th postnatal day, *n* = 6) in gerbils and compared the results to those of intramodiolar AAV injection in the adult gerbil (>8 weeks, 11.54 ± 0.63 weeks old, *n* = 11). AAV-PHP.B was chosen for its neurotropic potency (Deverman et al., 2016) and carried f-Chrimson (Mager et al., 2018) linked to the reporter enhanced yellow fluorescent protein (EYFP) under control of the human synapsin promoter. Approximately 10 weeks after injection (early postnatal: 10.92 ± 1.34 vs. adult: 9.25 ± 0.77 weeks, *p* = 0.42, Wilcoxon rank sum test), the SGN density and their expression of f-Chrimson-EYFP were analyzed by confocal microscopy of mid-modiolar cryosections immunolabeled for GFP and calretinin (Figure 1A and Supplementary Figure S1A) in both groups, regardless of the presence or absence of oABRs. For efficient analysis of the large number of neurons under study, a semi-automatic approach was developed to analyze the confocal sections. Specifically, we determined SGN density (i.e., the ratio between the number of SGN somata and the cross-sectional area of Rosenthal’s canal housing the spiral ganglion) and the density of GFP-positive SGN somata (GFP^+^ SGNs, Supplementary Figure S2). As illustrated in Supplementary Figure S1A, for a high proportion of cochleae included in this study the marker calretinin, calb2, did not support robust SGN detection. Therefore, SGN somata were manually detected based on background fluorescence in the GFP (green) channel by the experimenter using a touchscreen. Identified SGNs were then segmented automatically in a ∼20 ×∼20 μm window centered on the position marked by the user and the median green fluorescence of somatic SGN was measured. Second, we fitted a Gaussian mixture model with typically 1–3 components to the distribution of median somatic SGN fluorescence. The fluorescence threshold above which an SGN cell was considered being transduced was set as the mean + twice the standard deviation of the Gaussian with the lowest mean (considered as representing the background, see Supplementary Figure S2 and section “Materials and Methods” for more details).

The SGN density (Figure 1D) was similar between both groups across the 3 cochlear turns and amounted to an average per injected cochlea of 15.11 ± 1.15 and 15.69 ± 1.89 SGNs/10^4^ μm^2^ (*p* = 0.37, Wilcoxon rank sum test) for early postnatal (*n* = 6) and adult (*n* = 11) AAV injections, respectively. For both groups, the SGN density, averaged over the 3 cochlear turns, was significantly lower in the injected compared to the non-injected ear (Supplementary Figure S3A, quantified on a subset of animals for which histological data were available for both cochleae). The SGN density of early postnatally injected ears amounted to 15.11 ± 1.15 SGN/10^4^ μm^2^ compared to 22.94 ± 1.71 SGN/10^4^ μm^2^ in the contralateral non-injected ears (*n* = 6, *p* = 0.03, Wilcoxon signed rank test) and for adult injected ears: 14.81 ± 2.49 SGN/10^4^ μm^2^ vs. 20.28 ± 0.82 SGN/10^4^ μm^2^ in the contralateral non-injected ears (*n* = 7, *p* = 0.04, Wilcoxon signed rank test). While we anticipated neural loss for the injection into the adult Rosenthal canal probably due to the transient pressure increase during the injection (Wrobel et al., 2018), no such loss was previously reported for postnatally scala tympani injection into mouse ears (Keppeler et al., 2018; Mager et al., 2018). Moreover, the observed SGN densities were lower by 5–10 SGNs/10^4^ μm^2^ than reported in Wrobel et al. (2018), which we attribute to methodological differences (Supplementary Figure S1, see section “Materials and Methods,” histological quantification for more details).

We found transduced SGNs (GFP^+^ SGNs, Figure 1E) in all early postnatally injected ears (*n* = 6) and in ∼90% of adult injected ears (*n* = 11). The density of GFP^+^ SGNs was significantly higher across all cochlear turns for the early postnatally injected compared to the adult injected ears (Table 1).

**TABLE 1:** Density of GFP^+^ SGNs per cochlear turn and injection age (Wilcoxon rank sum test).

	GFP^+^ SGNs/10^4^ μ m^2^		*p*-value
	Early postnatal	Adult	
Apex	8.02 ± 2.16	2.64 ± 0.8	0.027
Middle	8.13 ± 1.45	1.53 ± 0.42	9.11 × 10^–4^
Base	10.37 ± 1.66	1.39 ± 0.43	9.11 × 10^–4^
Average of the 3 turns	8.96 ± 1.03	1.85 ± 0.51	9.11 × 10^–4^

Expressed as transduction rate, this corresponds to overall rates of 58.62 ± 4.46% and 12.99 ± 3.85% SGNs being transduced via the early postnatal and the adult injection, respectively. When only considering cochleae of adult injected gerbils from which an oABR (see below) could be measured, the transduction rate remained lower (19.27 ± 6.6%, 3.1 ± 1.14 GFP^+^ SGNs/10^4^ μm^2^) than found for early postnatal injection. Finally, investigating the presence of GFP^+^ SGNs in the non-injected cochleae revealed, in some cases, a spread of the virus to the contralateral ear upon early postnatal injection (Supplementary Figure S3B), whereas no GFP^+^ SGNs were found in ears contralateral to the adult injection side. The cochlear aqueduct and/or the endolymphatic duct were previously discussed as potential routes of viral spread via the cerebrospinal fluid space (Lalwani et al., 1996; Keppeler et al., 2018).

Next, we assessed the subcellular distribution of f-Chrimson-EYFP (Figure 1B,C) by measuring line profiles in GFP^+^ SGNs of injected cochleae (Figure 1F; i.e., GFP immunofluorescence intensity along a line orthogonal to the somatic SGN plasma membrane, measured from single confocal sections of SGNs immunolabeled for GFP and calretinin). The shorter distance between the intensity peak of the GFP immunofluorescence and the estimated position of the cell membrane (operationally defined as 30% of calretinin immunofluorescence rise) indicated a significantly greater plasma membrane expression of f-Chrimson-EYFP for early postnatally injected cochleae (0.88 ± 0.09 μm) compared to adult injected cochleae (1.37 ± 0.13 μm, *p* = 8.2 × 10^–3^, Wilcoxon rank sum test, Figure 1G). This result suggests a more efficient f-Chrimson-EYFP trafficking to the plasma membrane when transducing SGNs at an early age compared to adulthood.

Animals were tested for oABRs by inserting a 50 μm optical fiber coupled to an orange laser (λ = 594 nm) into their round window. Positive oABRs could be measured in 100% of the cochleae that received an early postnatal injection compared to 27.27% of adult injected cochleae. oABR traces, generally, showed heterogeneous morphology and were characterized by 2–4 positives waves (P1-P4; Figure 1H: traces with largest oABR amplitudes, Supplementary Figures S4A,B: all traces, black). Compared to animals that received an adult injection (*n* = 3/11), oABRs in early postnatally injected animals (*n* = 6/6) tended to have lower thresholds for light (Figure 1I, early postnatal injection: 1.2 ± 0.57 mW vs. adult injection: 5.33 ± 1.76 mW, we refrained from significance testing given the small number of oABR-positive adult injected animals), larger amplitudes (first wave: P_1_, Figure 1J, 2.17 ± 0.89 vs. 0.74 ± 0.37 μV; P_2_, Figure 1M, 2.59 ± 0.47 vs. 0.34 ± 0.28 μV), and shorter P_1_ latency (Figure 1K, 0.83 ± 0.07 vs. 1.38 ± 0.15 μV). The superior oABR responses of early postnatally injected animals are evident also from the growth of P_1_ amplitude with radiant flux (Figure 1L). This difference is in accordance with the larger number of transduced SGNs and the better f-Chrimson-EYFP SGN plasma membrane expression revealed by immunohistochemistry in early postnatally injected animals. The correlation between the histological and functional data is discussed below.

### Comparing AAV-PHP.B and AAV2/6 for Optogenetic Manipulation of Adult Gerbil SGNs

**FIGURE 2:**
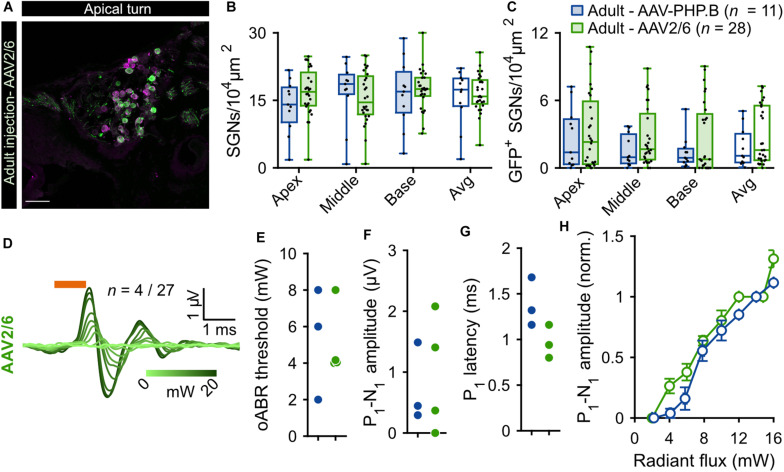
Comparison of f-Chrimson expression in SGNs and oABRs between AAV-PHP.B and AAV2/6 cochlear injections in adult gerbils. **(A)** Representative maximum projection of confocal images obtained from immunolabeled mid-modiolar cochlear cryosections (cochlear apical turn) following adult pressure cochlear injection of AAV2/6-f-Chrimson. GFP (green) marks transduced SGNs and calretinin (magenta) was used as a generic SGN marker (scale bar = 50 μm). **(B,C)** SGN density **(B)** and GFP-positive SGNs density **(C)** measured at the cochlear apical, middle and basal turn and average of the 3 turns for AAV-PHP.B (*n* = 11) and AAV2/6 (*n* = 28) f-Chrimson injected cochleae. Box plots show minimum, 25th percentile, median, 75th percentile, and maximum with individual data point overlaid. **(D)** Largest oABR recorded following AAV2/6 adult injected cochleae (λ = 594 nm, pulse duration = 1 ms, repetition rate = 10 Hz). oABRs could be recorded in 4 out of 27 gerbils. The color scale encodes the radiant flux. **(E–G)** oABR threshold **(E)**, P_1_-N_1_ amplitude (at 14 mW, **F**), and P_1_ latency (at 14 mW, **G**) measured from PHP.P (blue) and AAV2/6 (green) adult injected gerbils. **(H)** P_1_-N_1_ amplitude oABR growth function of AAV-PHP.B (blue) and AAV2/6 (green) injected gerbils. Amplitude were normalized relative to the amplitude at 14 mW. Average ± SEM. For all panels AAV-PHP.B data (blue) are replotted from Figure 1.

Given the limited transduction efficiency observed upon intramodiolar AAV administration into the adult cochlea, we compared the above described outcome of AAV-PHP.B (titer: 1.51 × 10^12^ genome copies/ml) to those obtained with a higher titer (9.9 × 10^12^ genome copies/ml) of AAV2/6, also carrying f-Chrimson-EYFP under the control of the human synapsin promoter. Figure 2 shows the AAV2/6 data (green) next to the AAV-PHP.B data (blue, replotted from Figure 1). AAV2/6 injected gerbils were slightly younger (9.88 ± 0.18 weeks old, *n* = 34) than the AAV-PHP.B group (11.54 ± 0.63 weeks old, *n* = 11, *p* = 0.02, Wilcoxon rank sum test). Representative confocal sections of mid-modiolar cryosections of AAV-PHP and AAV2/6 injected cochleae, immunolabeled for GFP (green) and calretinin (magenta), are shown in Figure 1A (lower panels) and Figure 2A, respectively. The SGN density in the injected ears (Figure 2B) was similar for both groups across the 3 cochlear turns and amounted to an average across the cochlea of 16.60 ± 0.72 SGNs/10^4^ μm^2^ for AAV2/6 vs. 15.69 ± 1.89 SGNs/10^4^ μm^2^ for AAV-PHP.B (*p* = 0.97, Wilcoxon rank sum test). At least one GFP^+^ SGN was found in 96.3% (26 out of 27) and 90.91% (10 out of 11) of mid-modiolar cryosections from AAV2/6 and AAV-PHP.B injected gerbil cochleae, respectively. The density of GFP^+^ SGNs (Figure 2C) tended to be higher for AAV2/6 (2.86 ± 0.44 SGNs/10^4^ μm^2^, transduction rate = 17.39 ± 2.64%) than for AAV-PHP.B (1.85 ± 0.51 SGNs/10^4^ μm^2^, transduction rate = 12.99 ± 3.85%), which however did not reach statistical significance (*p* = 0.33, Wilcoxon rank sum test). Despite the trend toward higher transduction rate, oABRs could only be recorded in 14.81% of AAV2/6 injected animals compared to 27.27% for AAV-PHP.B. The largest oABRs recorded are shown in Figure 2D for AAV2/6 and Figure 1H (lower panel) for AAV-PHP.B respectively. oABR thresholds (Figure 2E), P_1_-N_1_ amplitudes (Figure 2F), P_1_ latencies (Figure 2G), and P_1_-N_1_ growth functions (Figure 2H) were similar for both groups.

### Can Low Speed Injection Preserve SGNs Density?

**FIGURE 3:**
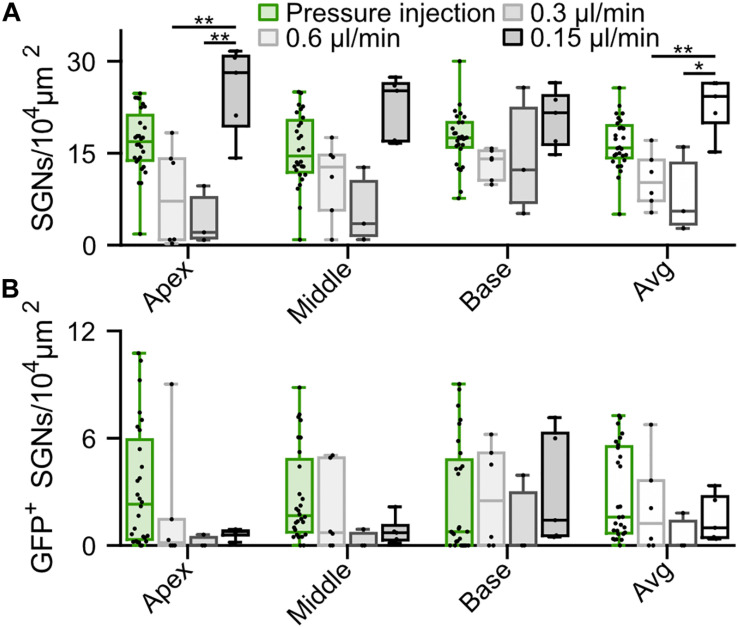
Comparison of f-Chrimson expression in SGNs following pressure or slow (0.6, 0.3, 0.15 μl/min) AAV2/6 intramodiolar injection. **(A,B)** SGN density **(A)** and density of GFP-positive SGNs **(B)** measured in the cochlear apical, middle, basal turns and averages across the 3 turns for pressure (green, *n* = 27) and slow injection: 0.6 μl/min (light gray, *n* = 6), 0.3 μl/min (gray, *n* = 3), 0.15 μl/min (black, *n* = 5). Kruskal-Wallis test followed by a Tukey’s multiple comparison test (*p ≤ 5 × 10^–2^, **p ≤ 10^–2^). Box plots show minimum, 25th percentile, median, 75th percentile, and maximum with individual data point overlaid.

In order to reduce the mechanical stress resulting from intramodiolar pressure injection and to better control the volume of virus suspension administered into the cochlea, we turned to a microsyringe pump-driven application. This allowed us to investigate the effect of different speeds of injection of 3 μl of AAV2/6 (titer = 9.9 × 10^12^ genome copies/ml; speed: 0.15 μl/min, *n* = 5; 0.3 μl/min, *n* = 3; 0.6 μl/min, *n* = 6). We administered the virus suspension via the intramodiolar route and, to promote the sealing of the pipette at the injection side, applied a droplet of hyaluronic acid hydrogel to the round window niche after insertion of the injection pipette into the drilled hole. 6 weeks after AAV2/6 administration (6.02 ± 0.41 weeks), immunohistochemistry, indeed, revealed a significantly higher SGNs density for injections at 0.15 μl/min compared to 0.3 and 0.6 μl/min (Figure 3A). This was evident for the cochlear apex (0.15 μl/min: 25.14 ± 3.29 SGNs/10^4^ μm^2^; 0.3 μl/min: 4.18 ± 2.75 SGNs/10^4^ μm^2^, *p* = 5.7 × 10^–3^; 0.6 μl/min: 7.98 ± 3.33 SGNs/10^4^ μm^2^, *p* = 8.9 × 10^–3^, Kruskal-Wallis test followed by a Tukey’s multiple comparison test) and when averaging the results of all three cochlear turns (0.15 μl/min: 22.77 ± 2.09 SGNs/10^4^ μm^2^; 0.3 μl/min: 8.09 ± 4.04 SGNs/10^4^ μm^2^, *p* = 1.8 × 10^–2^; 0.6 μl/min: 10.66 ± 1.81 SGNs/10^4^ μm^2^, *p* = 3.1 × 10^–4^; Kruskal-Wallis test followed by a Tukey’s multiple comparison test). Interestingly, the average SGN density following the slowest injection (22.77 ± 2.09 SGNs/10^4^ μm^2^) was similar to what observed for adult non-injected cochleae (20.28 ± 0.82 SGN/10^4^ μm^2^, Supplementary Figure S3, *p* = 0.22, Wilcoxon rank sum test). Nonetheless, the SGN density found with the slowest administration was not significantly greater than that of the pressure injection group (see above). The administration via the microsyringe pump did not offer an obvious advantage regarding the achieved transduction rate. Only few SGNs expressing f-Chrimson-EYFP (Figure 3B) tended to be found at the apex of the cochlea for the microsyringe pump administration for all three speeds in contrast to the pressure injected cochleae, while transduction rates were more comparable at the base of the cochlea. Too few positive oABRs were detected following the slow AAV2/6 injection to be quantified. Those we could record (Supplementary Figures S4D,E) were similar in shape, amplitude, and latency to oABRs following pressure injection and reported earlier in this study. These results suggest an advantage of the intramodiolar pressure injection in terms of number of transduced cells compared to the slow injection technique, at the expense of a mild SGNs loss. The SGNs loss seems to be avoidable by perfusing at ≤0.15 μl/min.

### Correlating oABRs to the Fraction of f-Chrimson Transduced SGNs

**FIGURE 4:**
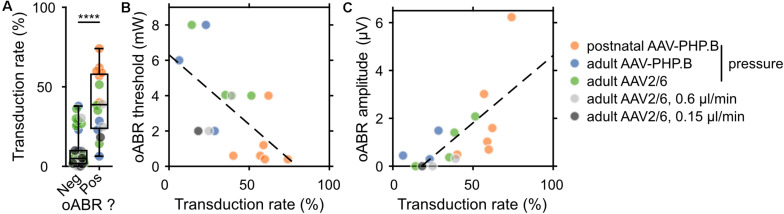
Correlating the rate of SGN transduction to f-Chrimson-mediated oABRs. **(A)** Transduction rate (defined as the ratio between the GFP^+^ SGN density and the SGN density) for gerbils lacking (Neg) or showing (Pos) an oABR. Wilcoxon rank sum test (****p ≤ 1 × 10^–4^). **(B,C)** Relationship between oABR threshold **(B)**, amplitude **(C)** and the transduction rate (TR). Dashed lines are fitted using linear models: **(B)** y (in mW) = –7.88 * TR + 6.3 (linear correlation test: *r* = –0.61, *p* = 0.011); **(C)** y (in μV) = 5.64 * TR – 1 (linear correlation test: *r* = 0.70, *p* = 0.0027). The color code for the different injection conditions is represented on the right of the figure.

Next, we related the transduction rate and oABR results for every animal included in this study, for which both the immunohistochemistry and oABR analysis were performed (postnatal AAV-PHP.B, *n* = 6; adult pressure-injected AAV-PHP.B, *n* = 11; adult pressure-injected AAV2/6, *n* = 27; adult 0.6 μl/min AAV2/6, *n* = 6; adult 0.3 μl/min AAV2/6, *n* = 6; adult 0.15 μl/min AAV2/6, *n* = 5). The average transduction rate (averaged across the cochlear turns, Figure 4A and pooled over all modes of injections and viral vectors) was significantly higher for cochleae from which an oABR could be recorded (39.43 ± 4.9%, *n* = 16) than for cochleae of gerbils without oABR response (9.84 ± 1.71%, *n* = 46, *p* = 2.73 × 10^–6^, Wilcoxon rank sum test). An oABR could already be observed for transduction rates of 6.35% (1.08 GFP^+^ SGN/10^4^ μm^2^). Surprisingly, also 19.57% of the negative animals had a transduction rate higher than 15%, suggesting a potential lack of sensitivity of the oABR elicited by optical stimulation from a 50 μm fiber measure as a screening method or that some transduced cells could have been not photosensitized (i.e., transduced but lacking proper membrane expression of the ChR, Keppeler et al., 2018; Wrobel et al., 2018). This later point was addressed by measuring the expression profile of f-Chrimson (Supplementary Figure S5A) relative to the estimated position of the cell membrane (i.e., 30% of calretinin immunofluorescence rise). The average distance between GFP peak and the estimated cell membrane position was similar between cochleae from which an oABR could be measured or not (Supplementary Figure S5B, negative oABR: *N* = 7 cochleae, *n* = 33 SGNs, 1.39 ± 0.15 μm; positive oABR: *N* = 5 cochleae, *n* = 38 SGNs, 1.37 ± 0.16 μm, *p* = 0.66, Wilcoxon rank sum test). We found a significant negative correlation between oABR threshold and transduction rate (TR, Figure 4B, *y* [in mW] = −7.88 ^∗^ TR + 6.3, *r* = −0.61, *p* = 0.011). Moreover, we obtained a significant positive correlation between the oABR amplitude and the transduction rate (Figure 4C, y [in μV] = 5.64 ^∗^ TR – 1, r = 0.70, *p* = 0.0027). Both regressions confirm the plausible hypothesis that the lowest threshold and biggest amplitude of oABRs are observed for cochleae with the highest proportion of SGNs expressing f-Chrimson.

## Discussion

Here, we used the Mongolian gerbil as preclinical model to further develop f-Chrimson-mediated optogenetic stimulation of SGNs. The Mongolian gerbil is of particular interest for auditory preclinical studies because its middle ear and cochlea are relatively large and, similarly to humans, hearing extends to the low frequency range (Huet et al., 2018). The achieved f-Chrimson expression enabled optical activation of the auditory pathway as indicated by recordings of oABRs. This set-up allowed us to test different time points and procedures for cochlear AAV administration as well as the impact of serotype and titer. Finally, we found a positive relationship between the strength of optogenetic activation of the auditory nerve and the rate of SGN transduction.

Different from previous studies, we systematically scrutinized viral transduction and membrane ChR expression of gerbil SGNs regardless of whether oABR could be recorded or not. We used f-Chrimson, a fast gating variant of the red-light activated ChR Chrimson because it has translational potential for optogenetic hearing restoration, similar to ChrimsonR used in the PIONEER clinical trial on optogenetic vision restoration (ClinicalTrials.gov, 2017, 03326336). Counting injected ears only, we analyzed a total of 17,749 SGNs from 66 cochleae. To do this efficiently, we developed a computer-assisted approach that also standardized and improved the determination of ChR–expressing SGNs. As calretinin was shown to be expressed only in a subset of SGNs (Petitpré et al., 2018; Shrestha et al., 2018; Sun et al., 2018; Wrobel et al., 2018) and calretinin staining was partially inconsistent in our samples (Supplementary Figure S1A), the detection of SGNs was more reliable when based on the background fluorescence in the GFP channel. Comparing to parvalbumin immunolabeling for detecting SGNs, we missed 5.56 SGN/10^4^μm^2^ (19.15%) by our approach based on background fluorescence. This likely led us to overestimate the transduction rate, such that we decided to also present number of transduced SGNs per unit area of the Rosenthal canal. Future studies should use more general neuronal markers as parvalbumin (Supplementary Figure S1B, Eybalin and Ripoll, 1990; Pack and Slepecky, 1995), β-III tubulin (Hallworth and Ludueña, 2000), or Na/K ATPase alpha 3 (McLean et al., 2009).

SGNs expressing f-Chrimson were found in the majority of the cochleae while an average transduction rate of 10% was also found in negative oABR cochleae, thus revealing a fair success of the viral gene transfer approaches used in this study. For comparison, Wrobel et al. (2018), using similar viral vector, titer, injection age, and injection route, did not find any transduced cells in the 4 negative oABR cochleae investigated for immunohistochemistry. In the current study, oABR could not be recorded in 19.5% of animals despite a SGN transduction rate higher than 15% (2.09 GFP^+^ SGNs/10^4^ μm^2^). This suggests a lack of sensitivity of the oABR in this particular setting, potentially owing to difficulties to find the optimal light projection from the small caliber (50 μm) optical fiber.

For the first time, to our knowledge, this study compared the optogenetic SGNs manipulation at an early postnatal stage (p8) and at the adult stage (>8 weeks) by cochlear AAV administration (AAV-PHP.B-hSyn-f-Chrimson). Similar to early postnatal AAV administration in mice (AAV2/6, Mager et al., 2018), we found oABRs with low thresholds in 100% of the early postnatally injected gerbils, high transduction rates (>50%), and a clear plasma membrane expression of f-Chrimson. The oABR threshold radiant flux was low (1.2 mW) similar to the previous findings in mice (0.5 mW, Mager et al., 2018). In contrast, adult injected cochleae showed a lower oABR success rate (∼30%), and, when present, smaller oABR amplitudes and higher threshold radiant flux (5.1 mW), as well as low transduction rate (∼10% for all cochleae and ∼20% for positive oABR cochleae). Those values are lower than previous reported on adult gerbil SGN using AAV2/6-CatCh (Wrobel et al., 2018) and the reasons for the difference are not obvious.

So why is the viral gene transfer into SGNs less efficient in the adult cochlea, despite direct intramodiolar injection close to the spiral ganglions rather than injecting more indirectly into the perilymph as for the immature scala tympani injections? Possible reasons for lower efficiency of transduction of older neurons include (*i*) a decrease of receptor-mediated endocytosis with age (Sato et al., 2001; Sasaki et al., 2002; Polinski et al., 2016) and (*ii*) a reduction of the neuronal surface availability due to the development of extracellular matrix and glial cells. Disruption of the perineural nets with hyaluronidase *in vivo* was shown to increase the motor cortex neurons transduction rate by 20% of 11 weeks old rats (Wanisch et al., 2013). Future studies should focus on optimizing virus tropism and ability to reach SGNs, e.g., by using rational design and directed evolution of AAVs (Dalkara et al., 2013) and investigate the effect of co-injection of drugs increasing virus access to the SGN surface by loosening up the extracellular matrix (Dalkara et al., 2009). In addition, SGNs of adult injected cochleae showed a reduced plasma membrane expression of f-Chrimson compared to those in early postnatally injected cochleae, suggesting a less efficient ChR trafficking in mature neurons. This result might not be generalizable, as Wrobel et al. (2018) reported excellent plasma membrane expression of CatCh in SGNs optogenetically modified at adult age (Supplementary Figure S1). Nonetheless, considering that the early postnatal injection occurs during the maturation of the SGNs, one might reason that f-Chrimson trafficking benefits from the overall dynamic cell sorting of ion channels in maturing SGNs (Kim and Rutherford, 2016). Strategies to enhance membrane trafficking of f-Chrimson in mature neurons include appending sequences to improve exit from the endoplasmic reticulum and trafficking to the plasma membrane of opsins (Gradinaru et al., 2010; Keppeler et al., 2018).

Next, highly neurotropic AAV-PHP.B (1.51 × 10^12^ genome copies/ml) and AAV2/6 at higher titer (9.9 × 10^12^ genome copies/ml) were compared for their efficiency to optogenetically modify adult SGNs using f-Chrimson. Cochleae injected with AAV2/6 showed a trend to a higher number of transduced SGNs compared to AAV-PHP.B, similar SGNs density but fewer oABR positive gerbils. A previous study using early postnatal injection into the mouse cochlea showed improved SGNs transduction using AAV-PHP.B compared to AAV2/6 both in terms of transduction rate and oABR success rate (Keppeler et al., 2018). Another study injecting adult gerbil cochleae with both vectors showed similar light threshold and strength of responses of the inferior colliculus to optogenetic SGN stimulation (Dieter et al., 2019). The common bias across those studies was to use AAV-PHP.B at lower titer than AAV2/6. Therefore, future work should compare these and other vectors at the same titer to decipher the one having the highest efficiency for transducing adult SGNs.

Finally, we showed that using a slow injection speed (≤0.15 μl/min) via a micropump allowed to preserve the SGN density compared to a pressure injection, for which a fraction of neurons was lost (Figure 3 and Wrobel et al., 2018). We propose that loss of SGNs results from cell death upon mechanical stress causing an immediate structural breakdown (Galluzzi et al., 2015) but cannot rule out other reasons. Nonetheless, using slow injection, only a small proportion of SGNs were transduced. These results suggest that a slow injection may be favorable to improve cell survival once efficient transduction is established. For example, future work could combine a slow injection with an additional cochlear vent for efficient exposure of the spiral ganglion to the virus suspension. This might increase the transduction rate while securing SGN survival. Approaches reported in the literature mostly aimed at administration to the perilymphatic space, combining, for example, round window injection with posterior semicircular canal fenestration (PSCC) or injection from the PSCC to the round window (Yoshimura et al., 2018; Talaei et al., 2019).

The present study highlights challenges that general cochlear gene therapy and specifically optogenetic approaches face to make their way to efficient and safe viral gene transfer into SGNs and expression of a transgene of interest. Future studies on developing an oCI toward clinical application should aim to efficiently and safely transduce SGNs with a red-shifted, fast-gating channelrhodopsin that traffics well to the plasma membrane for low-light intensity stimulation with a high temporal precision. Additionally, future studies should aim to develop red-light microscale emitters in order to develop fiber- or LED-based implementations of an optical cochlear implant.

## Materials and Methods

### Animals

All experiments were done in compliance with the German national animal care guidelines and were approved by the board for animal welfare of the University Medical Center Göttingen and the animal welfare office of the state of Lower Saxony (agreement 2014/1726 and 2017/2394). Experiments were performed on 69 Mongolian gerbils (*Meriones unguicalatus*) of both sexes. Early postnatal injections were performed at P8 and adult injections in animals older than 8 weeks (11.9 ± 0.82 weeks). Optically evoked auditory brainstem responses were recorded at least 8 and 4 weeks after injection for the early postnatal and adult injection, respectively.

### Virus Purification

The virus purification procedure is extensively described in Huet and Rankovic (2021). In brief, triple transfection of HEK-293T cells was performed using pHelper plasmid (TaKaRa/Clontech), trans-plasmid providing viral capsid PHP.B (generous gift from Ben Deverman and Viviana Gradinaru, Caltech, United States) and cis plasmid providing f-Chrimson. AAV2/6 AAV was purchased from UNC Vector Core. Viral particles were harvested 72 h after transfection from the medium and 120 h after transfection from cells and the medium. Precipitation of the viral particles from the medium was done with 40% polyethylene glycol 8000 (Acros Organics, Germany) in 500 mM NaCl for 2 h at 4°C and then after centrifugation at 4,000 g for 30 min combined with cell pellets (already processed by salt-activated nuclease (SAN, Arcticzymes, United States) for additional 30 min SAN incubation at 37°C. Afterward, the cell lysates were clarified by centrifugation at 2,000 *g* for 10 min and then purified over iodixanol (Optiprep, Axis Shield, Norway) step gradients (15, 25, 40, and 60%) (Zolotukhin et al., 1999; Grieger et al., 2006) at 58,400 rpm for 2.25 h. Finally, viral particles were concentrated using Amicon filters (EMD, UFC910024) and formulated in sterile phosphate-buffered saline (PBS) supplemented with 0.001% Pluronic F-68 (Gibco, Germany). Virus titers were obtained according to manufacturer’s instructions by determining the number of DNase I resistant vg using qPCR (StepOne, Applied Biosystems) and AAV titration kit (TaKaRa/Clontech). Purity of produced viruses was routinely checked by silver staining (Pierce, Germany) after gel electrophoresis (Novex^TM^ 4–12% Tris-Glycine, Thermo Fisher Scientific) according to manufacturer’s instruction. The presence of viral capsid proteins was positively confirmed in all virus preparations. Viral stocks were kept at −80°C until experiment day.

### Virus Injection

Approximately 3 μl of virus were injected per cochlea via quartz micropipettes (tip diameter ∼20 μm, Science products; pulled with a P-2000 laser puller, Sutter Instruments) connected to a pressure microinjector (100–125 PSI, PLI-100 pico-injector, Harvard Apparatus) or a microsyringe pump (Micro4, World Precision Instruments). In this study, 2 capsids containing the same plasmid were compared: PHP.B (1.51 × 10^12^ genome copies/ml) and AAV 2.6 (9.9 × 10^12^ genome copies/ml). The plasmid contained the opsin f-Chrimson linked to the reporter protein enhanced yellow fluorescent protein under control of the promoter human synapsin.

### Early Postnatal Injection of the Cochlea

Injections in the scala tympani of the left cochlea were performed at P8 essentially as described in Huet and Rankovic (2021). In brief, anesthesia was obtained by isoflurane (5% for anesthesia induction, 1–2% for maintenance, frequent testing of the absence of hind-limb withdrawal reflex) and analgesia using subdermal injection of buprenorphine (0.1 mg/kg body weight) and carprofen (5 mg/kg body weight, repeated 48 h after procedure). Body temperature was maintained warm by placing the animal on a remote-controlled custom-build heating blanket. Following a retro-auricular approach, the facial nerve was exposed in order to determine where to puncture the cartilaginous bulla with the injection pipette and target the scala tympani where virus suspension (1–1.5 μl) was injected. Following the injection, the surgical situs was closed by suturing the skin.

### Adult Injection of the Cochlea

Adult injections were performed as described in Wrobel et al. (2018). In brief, anesthesia was obtained by isoflurane (5% for anesthesia induction, 1–2% for maintenance, frequent testing of the absence of hind-limb withdrawal reflex) and proper analgesia by subdermal injection of buprenorphine (0.1 mg/kg body weight) and carprofen (5 mg/kg body weight, repeated 48 h after procedure). Body temperature was maintained warm by placing the animal on a remote-controlled custom-build heating blanket. The left cochlea was exposed following a retro-auricular approach and a bullostomy. A 200 μm hole was drilled into the basal modiolus using a Kflex dental file (no. 15) bypassing the *scala tympani*. Then, 3 μl of virus suspension was injected through the drilling hole. In the case of the slow injection using the injection pump, the sealing of the pipette tip to the inner ear was guaranteed by sealing the surrounding of the pipette tip with hyaluronic acid hydrogel (1%). After the injection, the opened bulla was covered by retro-auricular autologous connective tissue and the skin sutured.

### Optically Evoked Auditory Brainstem (oABR) Response

oABRs were recorded under anesthesia using isoflurane (5% for anesthesia induction, 1–2% for maintenance, frequent testing of the absence of hind-limb withdrawal reflex) and proper analgesia by subdermal injection of buprenorphine (0.1 mg/kg body weight) and carprofen (5 mg/kg body weight). The negative impact of isoflurane showed on acoustically evoked ABR (Ruebhausen et al., 2012) was considered to be of limited if of any relevance on optogenetically evoked ABR, as (*i*) the optogenetic activation of the SGNs do not rely on glutamate released by the IHCs and (*ii*) the oABR threshold was determined mostly on wave I. The body temperature was maintained at ∼37°C by a remote-controlled custom-made heating pad. The round window of the cochlea was exposed following a retro-auricular approach as described above for the adult injection preparation. Light delivery into the cochlea was ensured by inserting in the round window a 50 μm optical fiber coupled to a 594 nm laser (OBIS LS OPSL, 100 mW, Coherent Inc., Santa Clara, CA, United States). Laser power was calibrated prior to each experiment using a laser power meter (LaserCheck, Coherent Inc., Santa Clara, CA, United States). oABRs were recorded using 3 needle electrodes inserted bellow the left pinna, at the vertex and on the back of the animal near the tail. oABRs were amplified using a custom-made physiological amplifier, sampled at a rate of 50 kHz (NI PCI-6229, National Instrument), digitally filtered (300–3,000 Hz), and averaged 1,000 times. Stimulus generation and data acquisition were made using a custom-written software (MATLAB, MathWorks) employing National Instrument data acquisition cards in association with custom-build laser-controller. Recordings were performed in a soundproof chamber (IAC Acoustics, Illinois, United States). oABR traces were analyzed offline using a custom-made software (MATLAB, MathWork) allowing detection of oABRs waves I, II, and III. The oABRs threshold was determined as the lowest light intensity for which one of the 3 waves was reliably visible. The latency of a given wave was defined as the delay between the stimulus onset and the wave of interest.

### Immunohistochemistry and Confocal Microscopy

Following puncture of the oval and round windows, cochleae were fixed using formaldehyde (4%) in PBS for 1 h, decalcified using EDTA (0.12 M) for 7 days, and cryosectioned. Sections were then incubated for 1 h in donkey serum dilution buffer, overnight at 4°C in primary antibody solution, and 1 h at room temperature in secondary antibody solution. For injected cochleae, the following primary antibody was used: goat anti-Calretinin (CG1, Swant, 1:300) and the following AlexaFluor-labeled secondary antibodies were used: rabbit anti-GFP Alexa fluor 488 (A-21311, Thermo Fisher Scientific, 1:500), donkey anti-goat 568 IgG (H + L) (A-11057, Invitrogen). For some control cochleae, anti-calretinin primary antibody was replaced by guinea pig anti-parvalbumin (195004, Synaptic Systems, 1:300) and goat anti-guinea pig 568 IgG (H + L) (A1107, Thermo Fisher Scientific, 1:200). Confocal images were collected using either a SP5 microscope (Leica, Hamburg, Germany) or a LSM510 (Zeiss, Jena, Germany).

### Histological Quantification

The quantification of the number of SGNs was made using a self-written algorithm (MATLAB, MathWorks) allowing a computer assisted thresholding. Calretinin initially employed in this study as a neuronal marker was recently shown to not be expressed in every SGNs subtypes (Petitpré et al., 2018; Shrestha et al., 2018; Sun et al., 2018). Additionally in a high proportion of the samples, calretinin immunofluorescence was barely visible (Supplementary Figure S1A). Therefore, SGN somas were manually detected in the background fluorescence of GFP immunolabeling (Supplementary Figures S1A, S2A,B). The detection was made using a touch screen. On additional control cochleae (Supplementary Figures S1B,C, *n* = 11) stained for the neuronal marker parvalbumin, we quantified to miss 19.15% of the SGNs by using the background fluorescence of GFP immunolabeling (23.43 ± 1.71 SGNs/10^4^ μm^2^) compared to parvalbumin immunolabeling (29.03 ± 1.49 SGNs/10^4^ μm^2^, *p* = 9.77 × 10^–4^, Wilcoxon signed rank test).

In a 22.72 × 22.72 μm (SP5, Leica) or 18.64 × 18.64 μm (LSM510, Zeiss) window centered on the cell position (defined by the user in the first step), in an iterative process SGN somas were segmented using a custom-written function based on the Otsu’s thresholding method and the segmented object was validated using 2 criterions: (*i*) superimposition of the segmented object and the position given by the user; (*ii*) segmented object centroid not further than 3.78 μm from the position given by the user (Supplementary Figure S2C_1__–__4_). For each SGN median somatic GFP immunofluorescence were measured. Using a Gaussian mixture model (Supplementary Figure S2D, number of components adjusted by the user, typically between 1 and 3) fitted to the GFP immunofluorescence distribution, a threshold defined as the mean plus 2 times the standard deviation of the distribution with the lowest mean (i.e., background fluorescence of GFP immunolabeling) was used to define GFP positive SGNs (Supplementary Figure S2E).

### Data Analysis

Data was analyzed using Matlab (MathWorks). Averages were expressed as mean ± SEM in text and figures. Data were tested for normality using a Jarque-Bera test. As all data in this study were not normally distributed, only non-parametric statistical tests were used. For statistical comparison between two independent groups, a Wilcoxon rank sum test was used; for statistical comparison between more than two independent groups, a Kruskal-Wallis test followed by a Tukey’s multiple comparison test were used; for statistical comparison between two paired groups, a Wilcoxon signed rank test was used.

## Data Availability Statement

The original contributions presented in the study are included in the article/Supplementary Material, further inquiries can be directed to the corresponding author/s.

## Ethics Statement

The animal study was reviewed and approved by the Niedersächsisches Landesamt für Verbraucherschutz und Lebensmittelsicherheit.

## Author Contributions

VR generated virus and performed virus injection of early postnatal animals together with TD. TD performed adult virus injection and oABR recordings. TD and AH performed confocal microscopy. AH and AT developed the cell counting tool used for the histological quantification. AH analyzed the data and prepared figures. TD, AH, and TM designed the study, prepared the manuscript and all authors contributed.

## Conflict of Interest

TM is a co-founder and CEO of OptoGenTech company. The remaining authors declare that the research was conducted in the absence of any commercial or financial relationships that could be construed as a potential conflict of interest.

## Supplementary Material

Supplementary Figure 1 Advantages and limitations of using background fluorescence of the GFP channel to detect and count SGN somas. **(A,B)** Immunolabelled mid-modiolar cryosections stained for GFP/calretinin **(A)** and GFP/parvalbumin **(B)**. Note that more SGN somas can be detected from GFP channel than from calretinin. In contrast, SGN somas are easier to detect using parvalbumin immunolabeling than background fluorescence of GFP **(B)**. **(C)** Quantification of the SGN density measure from GFP and parvalbumin (PV) immunolabeling from 11 control cochlear turns. Wilcoxon signed rank test (^****^*p* ≤ 1 × 10^–4^).

Supplementary Figure 2 GFP-positive SGN detection based on a computer-assisted thresholding approach. **(A,B)** From the maximum projection of the GFP immunofluorescence **(A)**, the users clicked on the SGNs position using a touchpad **(B)**, green dots correspond to SGNs positions). **(C)** Example of automatically segmented SGNs in a 22.72 μm window centered on the position given by the user in **(B)**. Scale bar = 5 μm. **(D)** Distribution of the immunofluorescence measured for all SGNs detected by the user in **(B)**. A Gaussian mixture model is fitted in order to define the threshold for GFP-positivity as the average + 2 x the standard deviation of the distribution with the smallest mean. **(E)** GFP-positive SGNs defined as the SGNs for which the average brightness is higher than the threshold defined in **(D)**. Scale bar in **(A,B,E)** = 50 μm.

Supplementary Figure 3 Non-injected ear SGNs density and GFP^+^ SGN density following early postnatal (*n* = 6) and adult (*n* = 7) injected cochleae using AAV-PHP.B-f-Chrimson. **(A,B)** SGN density **(B)** and GFP positive SGNs density **(C)** measured at the cochlear apical, middle and basal turn and average of the 3 turns following early postnatal (orange, *n* = 6) and adult (blue, *n* = 7) injected cochleae of AAV-PHP.B-f-Chrimson. Wilcoxon signed rank test (^∗^*p* ≤ 1 × 10^–2^). Box plots show minimum, 25th percentile, median, 75th percentile, and maximum with individual data point overlaid.

Supplementary Figure 4 oABR (black, 14 mW radiant flux, 10 Hz repetition rate, 1 ms light pulse, 1,000 repetitions) and acoustically evoked ABR (gray, 300 μs acoustic click, 50 dB SPL, 10 Hz, 1,000 repetitions) traces for all positive oABR animals in this study, divided by injection group: **(A,B)** Early postnatal **(A)** and adult **(B)** pressure injected cochlea using AAV-PHP.B-f-Chrimson; **(C–E)** Adult pressure **(C)**, slow injection 0.6 μl/min **(D)** and slow injection 0.15 μl/min **(E)** injected cochlea using AAV2/6-f-Chrimson.

Supplementary Figure 5 Subcellular distribution of f-Chrimson-EYFP in SGNs from cochleae lacking or showing an oABR following an AAV2/6-f-Chrimson intramodiolar injection. **(A)** Normalized line profiles were measured on single SGN somata for calretinin (calb2, dashed lines) and GFP (plain lines) immunolabeling at the 3 cochlear turns, normalized and aligned at 30% of calretinin rise for SGN somatas from gerbils lacking (Neg, black, *N* = 7 cochleae, *n* = 33 SGNs) or showing (Pos, green, *N* = 5 cochleae, *n* = 38 SGNs) an oABR following an AAV2/6 injection at adult age. **(B)** Quantification of the distance between the estimated cell membrane (i.e., 30% of calretinin rise) and the GFP maximum peak of the line profile for negative (black) and positive (green) oABRs cochleae (data presented in **A**). Box plots show minimum, 25th percentile, median, 75th percentile, and maximum.
