# Supplementary figures and images for: Developing Fast, Red-Light Optogenetic Stimulation of Spiral Ganglion Neurons for Future Optical Cochlear Implants
Source: Front Mol Neurosci. 2021 Mar 11;14:635897. doi: 10.3389/fnmol.2021.635897 (PMC7991399; doi:10.3389/fnmol.2021.635897)

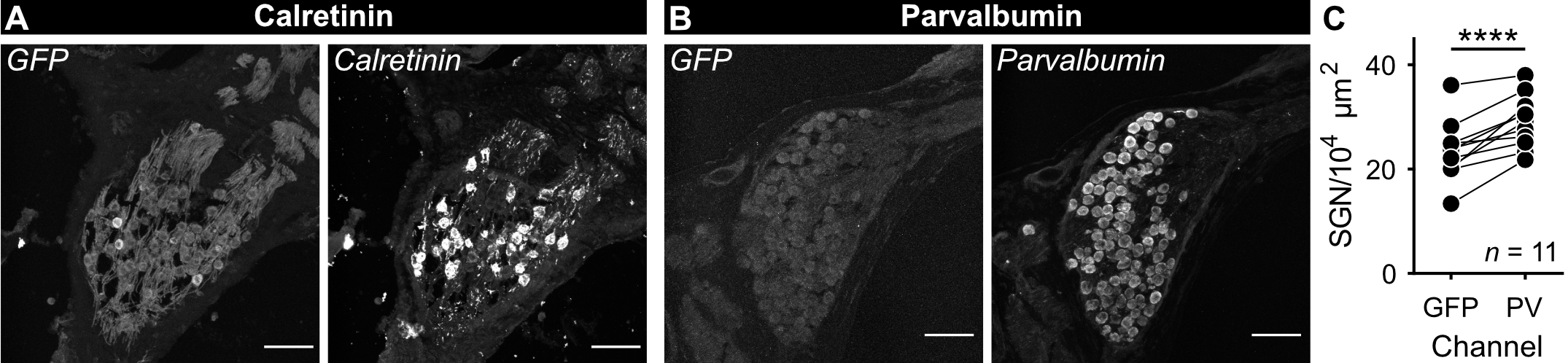

Supplement: Supplementary Figure 1 — Advantages and limitations of using background fluorescence of the GFP channel to detect and count SGN somas. (A,B) Immunolabelled mid-modiolar cryosections stained for GFP/calretinin (A) and GFP/parvalbumin (B). Note that more SGN somas can be detected from GFP channel than from calretinin. In contrast, SGN somas are easier to detect using parvalbumin immunolabeling than background fluorescence of GFP (B). (C) Quantification of the SGN density measure from GFP and parvalbumin (PV) immunolabeling from 11 control cochlear turns. Wilcoxon signed rank test (****p ≤ 1 × 10^–4^). [file Image_1.TIFF]

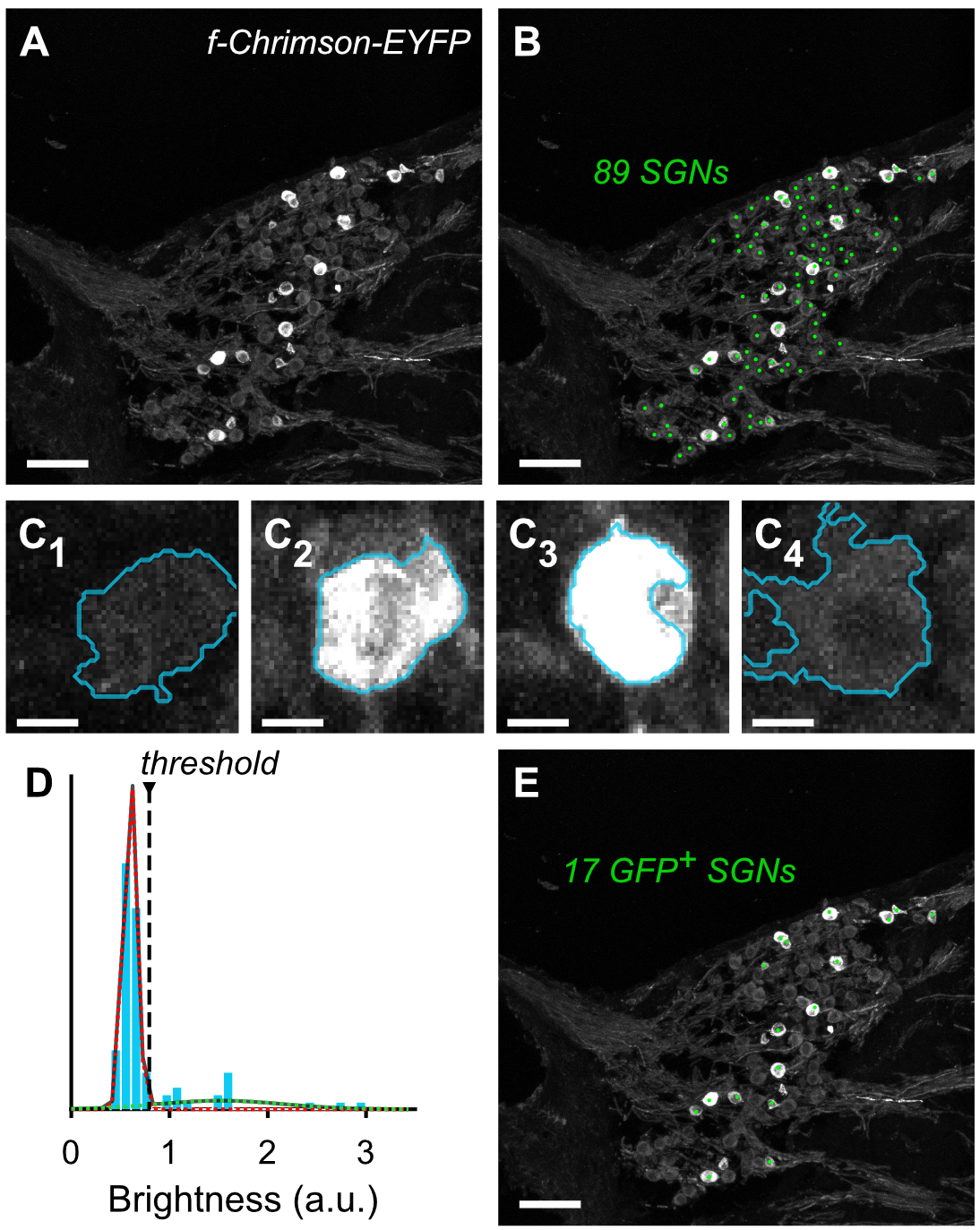

Supplement: Supplementary Figure 2 — GFP-positive SGN detection based on a computer-assisted thresholding approach. (A,B) From the maximum projection of the GFP immunofluorescence (A), the users clicked on the SGNs position using a touchpad (B), green dots correspond to SGNs positions). (C) Example of automatically segmented SGNs in a 22.72 μm window centered on the position given by the user in (B). Scale bar = 5 μm. (D) Distribution of the immunofluorescence measured for all SGNs detected by the user in (B). A Gaussian mixture model is fitted in order to define the threshold for GFP-positivity as the average + 2 x the standard deviation of the distribution with the smallest mean. (E) GFP-positive SGNs defined as the SGNs for which the average brightness is higher than the threshold defined in (D). Scale bar in (A,B,E) = 50 μm. [file Image_2.TIFF]

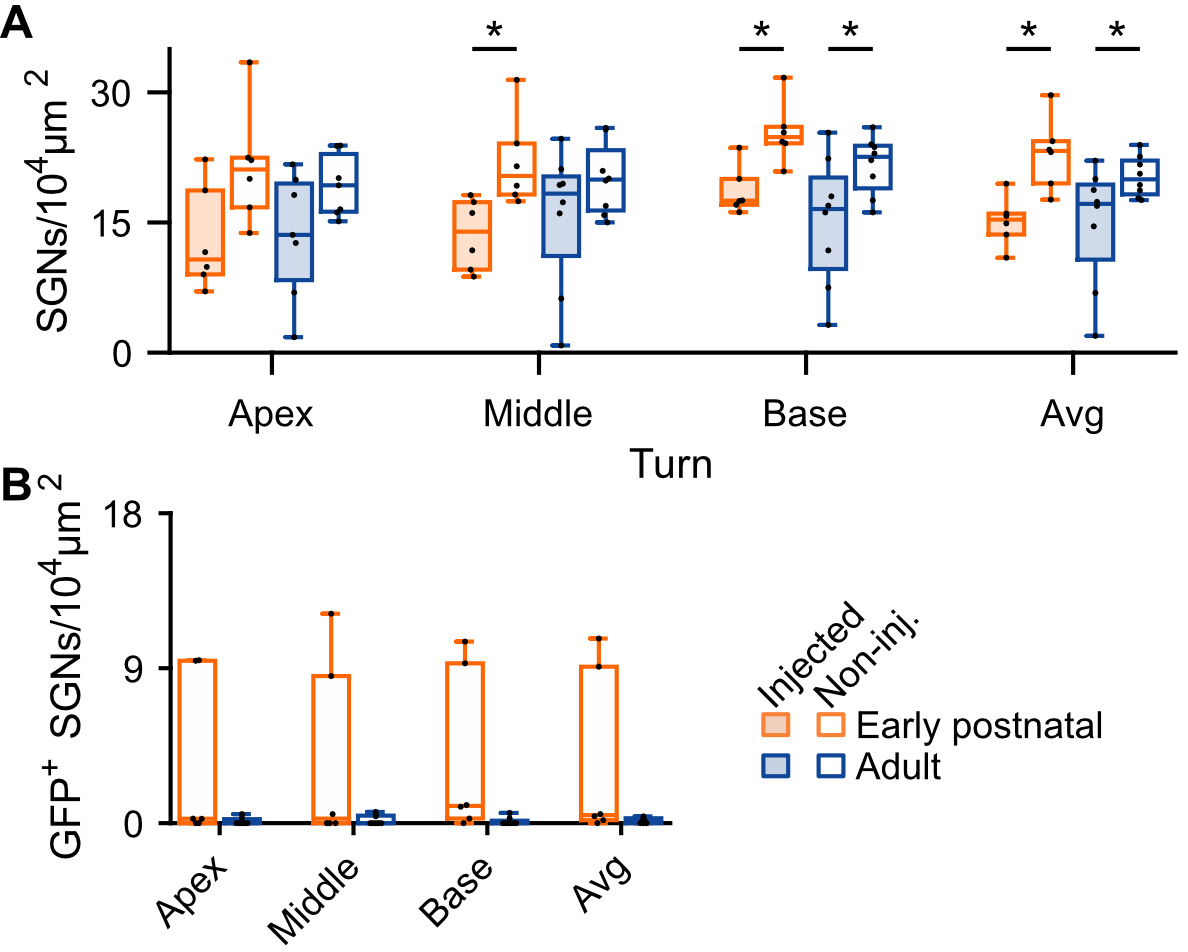

Supplement: Supplementary Figure 3 — Non-injected ear SGNs density and GFP+ SGN density following early postnatal (n = 6) and adult (n = 7) injected cochleae using AAV-PHP.B-f-Chrimson. (A,B) SGN density (B) and GFP positive SGNs density (C) measured at the cochlear apical, middle and basal turn and average of the 3 turns following early postnatal (orange, n = 6) and adult (blue, n = 7) injected cochleae of AAV-PHP.B-f-Chrimson. Wilcoxon signed rank test (∗p ≤ 1 × 10^–2^). Box plots show minimum, 25th percentile, median, 75th percentile, and maximum with individual data point overlaid. [file Image_3.TIFF]

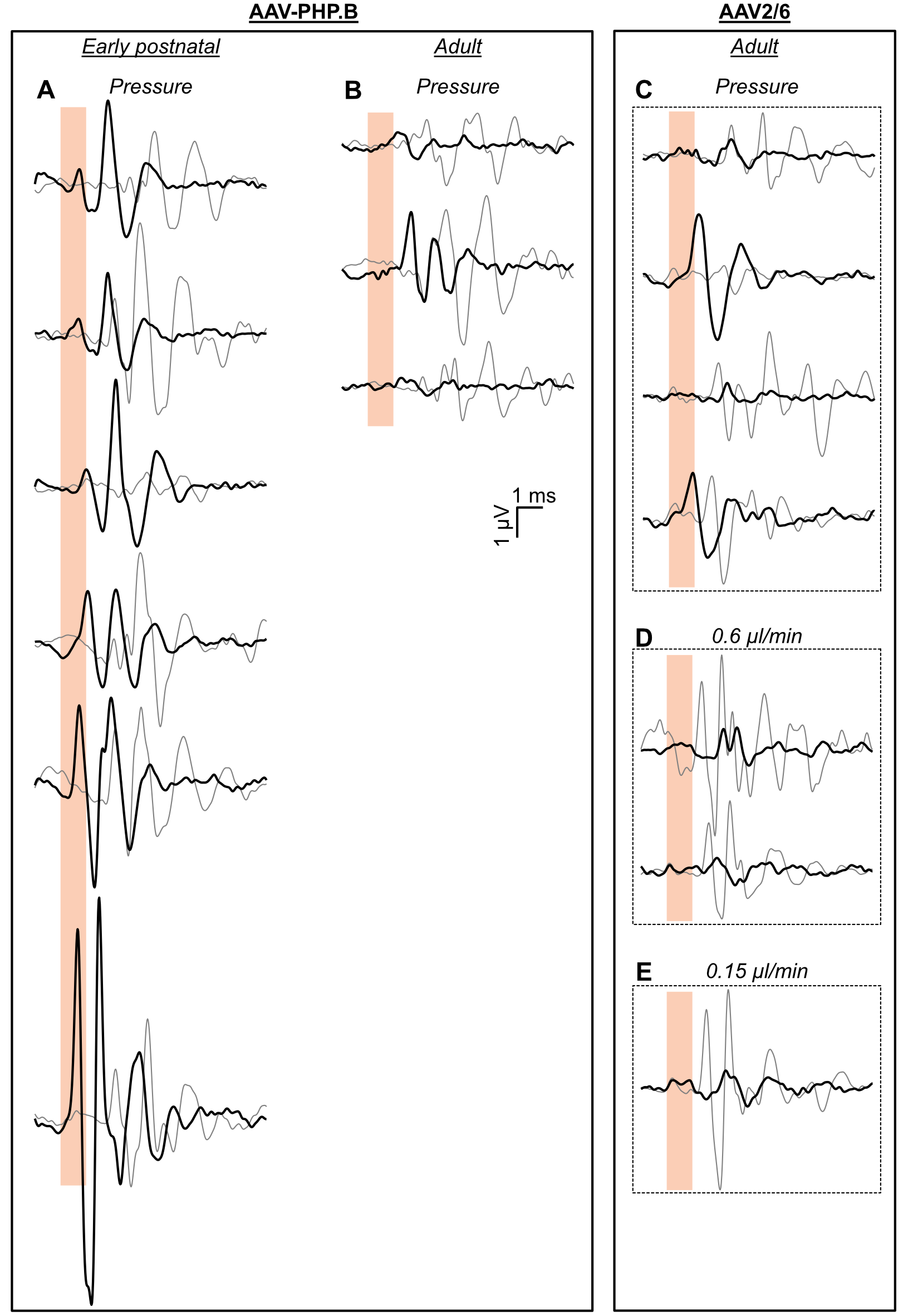

Supplement: Supplementary Figure 4 — oABR (black, 14 mW radiant flux, 10 Hz repetition rate, 1 ms light pulse, 1,000 repetitions) and acoustically evoked ABR (gray, 300 μs acoustic click, 50 dB SPL, 10 Hz, 1,000 repetitions) traces for all positive oABR animals in this study, divided by injection group: (A,B) Early postnatal (A) and adult (B) pressure injected cochlea using AAV-PHP.B-f-Chrimson; (C–E) Adult pressure (C), slow injection 0.6 μl/min (D) and slow injection 0.15 μl/min (E) injected cochlea using AAV2/6-f-Chrimson. [file Image_4.TIFF]

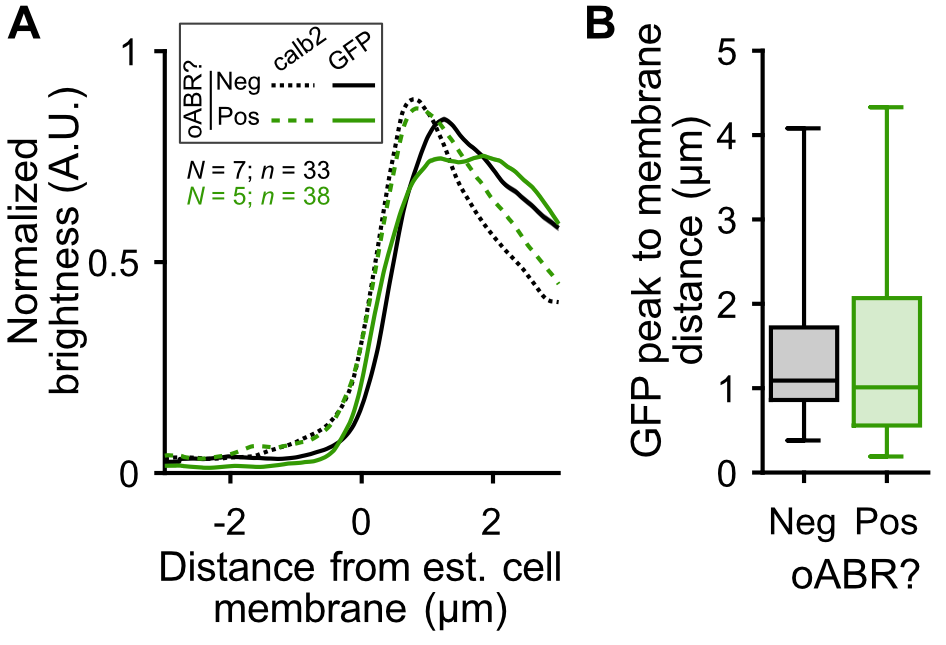

Supplement: Supplementary Figure 5 — Subcellular distribution of f-Chrimson-EYFP in SGNs from cochleae lacking or showing an oABR following an AAV2/6-f-Chrimson intramodiolar injection. (A) Normalized line profiles were measured on single SGN somata for calretinin (calb2, dashed lines) and GFP (plain lines) immunolabeling at the 3 cochlear turns, normalized and aligned at 30% of calretinin rise for SGN somatas from gerbils lacking (Neg, black, N = 7 cochleae, n = 33 SGNs) or showing (Pos, green, N = 5 cochleae, n = 38 SGNs) an oABR following an AAV2/6 injection at adult age. (B) Quantification of the distance between the estimated cell membrane (i.e., 30% of calretinin rise) and the GFP maximum peak of the line profile for negative (black) and positive (green) oABRs cochleae (data presented in A). Box plots show minimum, 25th percentile, median, 75th percentile, and maximum. [file Image_5.TIFF]
